# 2,5-PRODAN Derivatives as Highly Sensitive Sensors of Low Solvent Acidity

**DOI:** 10.3390/molecules19056415

**Published:** 2014-05-20

**Authors:** Alexandra H. Yoon, Laura C. Whitworth, Joel D. Wagner, Christopher J. Abelt

**Affiliations:** Department of Chemistry, College of William and Mary, Williamsburg, VA 23187-8795, USA

**Keywords:** H-bonding, sensor, fluorescence, quenching

## Abstract

Two 5-acyl-2-dimethylaminonaphthalene derivatives, one with a propionyl group and the other with a fused cyclohexanone ring, are investigated as sensors of H-bond-donating ability in protic solvents of low solvent acidity. Their fluorescence is highly quenched in protic solvents, and the quenching order of magnitude is linearly related to the H-bond-donating ability of the solvent as quantified by the solvent acidity (SA) scale. As the solvent acidity increases from 0.15 to 0.40, the fluorescence for both is quenched by more than a factor of ten; thus, they are extremely sensitive sensors of the hydrogen-bond-donating ability in this weakly acidic range. Preferential solvation studies suggest that quenching occurs from a doubly H-bonded excited state.

## 1. Introduction

The development of molecular sensors is an active field of research. Molecules can act as sensors when they have a measurable physical quantity that is responsive to some external stimulus [1]. Molecular sensors find most of their use in biological systems and materials. Fluorescent compounds are often recruited as sensors. Fluorescence offers a number of quantifiable outputs; namely, intensity, position and lifetime. All of these can be affected by the interactions between the excited sensors and their immediate environments.

One molecule that has received particular attention as a fluorescent sensor is 6-propionyl-2-dimethyaminonaphthalene (PRODAN). Weber and Farris prepared this compound as a molecular reporter for the binding site of bovine serum albumin [2]. The charge-transfer nature of the excited state results in strong solvatochromism. Ideally, the polarity of the microenvironment can be derived from the position of the fluorescence band. However, solvents can perturb electronically excited states through several modes of interactions besides polarity. Generally these modes are divided into H-bond donating (acidity) and accepting (basicity) ability in addition to some polarity measure(s) [3]. In the Kamlet-Taft approach, solvent properties are quantified by the α, β and π* parameters, respectively [4]. In the Catalán approach these parameters are called solvent acidity (SA), solvent basicity (SB) and solvent polarity/polarizability (SPP), although the latter polarity term has been further divided into polarizability (SP) and dipolarity (SdP) [5,6]. Solvatochromic analysis of PRODAN's emission shows that H-bonding can lead to significant red-shifting of the fluorescence [7,8]. Thus, the position of the emission band depends on both polarity and H-bonding, and deducing the magnitude of the micropolarity from the band position is not justified except with aprotic environments [6,9].

**Figure 1:**
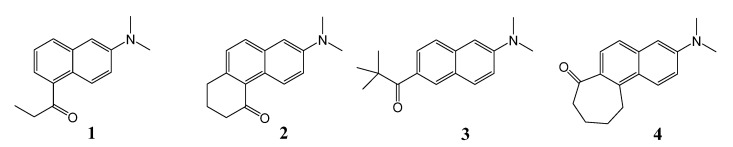
Structures of 2,5-PRODAN derivatives **1** and **2** and carbonyl-twisted 2,6-PRODAN derivatives **3** and **4**.

Sensors of solvent acidity are relatively rare. Recently we have shown that several PRODAN derivatives possessing carbonyl groups that are twisted out-of-plane with the naphthalene ring are good sensors for the H-bonding ability of alcohol solvents. Specifically, with derivatives **3** and **4** (Figure 1) fluorescence quenching can be correlated with the solvent acidity using Catalán’s SA parameter [10]. These sensors operate over a wide range of high acidities. They suffer quenching of nearly two orders of magnitude going from isopropanol (SA = 0.283) to water (SA = 1.062). We have used **3** and **4** as sensors for the local solvent acidities of their aqueous complexes with β-cyclodextrin [11]. For environments with extremely low solvent acidities 4'-dialkylamino-3-hydroxyflavones are effective sensors [12]. Even weakly H-bond-donating solvents (SA < 0.04) will form H-bonds with these molecules, and the proportion of H-bonded and free molecules can be determined from the ratio of the intensities of the two emission bands.

The regioisomeric PRODAN derivatives **1** and **2** (Figure 1) display excited-state charge-transfer behavior similar to PRODAN [13]. As a result, the fluorescence maxima are also shifted to the red in solvents of increasing polarity. As with **3** and **4**, the excited-states are strongly affected by specific interactions with protic solvents resulting in extremely efficient quenching.

The difference in the relative positioning of the amino and carbonyl groups in **1** and **2** *vs.* the PRODAN derivatives affects the excited-state structures. In **1** and **2** the CT state has the same electronic configuration as the initial locally excited-state, whereas PRODAN and **3** and **4** undergo internal conversion to a CT state with a different electronic configuration. This difference appears to be unimportant for the quenching mechanism in protic solvents. In this paper we show that **1** and **2** are very sensitive sensors of H-bond-donating ability in the low-to-middle range of solvent acidity. They are complementary to the acidity sensors **3** and **4**.

## 2. Results and Discussion

### 2.1. Fluorescence Quenching in Alcohols

**Figure 2:**
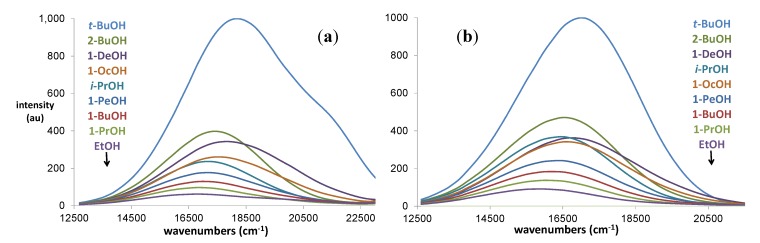
(**a**) Fluorescence of **1** in various alcohols (λ_ex_ = 405 nm); (b) Fluorescence of **2** in various alcohols (λ_ex_ = 405 nm).

The fluorescence of **1** and **2** in a series of nine alcohols is shown in Figure 2. These alcohols include 1°, 2° and 3° structures. They range in solvent acidity from a high of 0.4 for ethanol to a low of 0.145 for *t*-butanol (Table 1). In contrast, their absorption spectra are little affected by the solvent (*vide infra*). Both **1** and **2** show remarkable fluorescence quenching over this range of solvents. The fluorescent intensity generally follows the solvent acidity parameter, but not any of the remaining parameters. The spectra in *t*-butanol, decanol and octanol, all solvents with low acidities, show tailing to high wavenumbers. While 2-butanol also has low acidity, it gives rise to a larger peak intensity than the latter three solvents, but no tail.

**Table 1:** Catalán solvent parameters for the nine alcohols used in Figure 2.

	*t*-BuOH	2-BuOH	1-DeOH	1-OcOH	*i*-PrOH	1-PeOH	1-BuOH	1-PrOH	EtOH
SP	0.632	0.656	0.722	0.633	0.713	0.687	0.674	0.658	0.633
SdP	0.732	0.706	0.383	0.808	0.454	0.587	0.655	0.748	0.783
SA	0.145	0.221	0.259	0.283	0.299	0.319	0.341	0.367	0.400
SB	0.928	0.888	0.912	0.830	0.923	0.860	0.809	0.782	0.658

**Figure 3:**
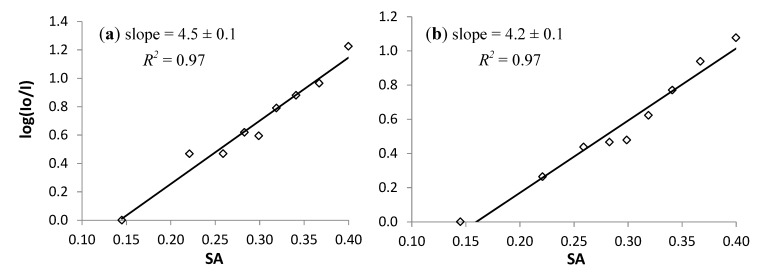
(**a**) Plot of log(I_max_/I_solvent_) *vs.* SA for **1**; (**b**) Plot of log(I_max_/I_solvent_) *vs.* SA for **2**.

The quenching behavior of **1** and **2** is characterized through the integrated fluorescent intensities. The quenching order of magnitude in each of the solvents is determined as log(I_max_/I_solvent_) where I_max_ is the largest I_solvent_ value for that compound. The intensities have been adjusted to account for differences in the refractive indicies and molar absorptivities at 405 nm [14]. The plots of the quenching order of magnitude *versus* Catalán’s solvent acidity for compounds **1** and **2** are shown in Figure 3. Both compounds show nearly linear quenching with this series of monoalkanols. The magnitude of the slopes of these plots is a measure of the sensitivity of the response to solvent acidity. The linearity of the plots is another important characteristic for a potential sensor. The slopes for these plots are both over four with *R^2^* values of 0.97. By comparison, the carbonyl-twisted PRODAN derivatives **3** and **4** show smaller slopes (~2) in their plots and quenching over a higher SA range (0.3 to 1.1). For both **1** and **2** the fluorescence intensities decrease over this very small range of solvent acidities by slightly more than one order of magnitude. With better H-bond-donating solvents such as methanol quenching is essentially complete.

The plots in Figure 3 suggest that quenching is related to solvent acidity. Quenching does not correlate well with other solvent characteristics. The lack of correlation is demonstrated through multilinear regression analysis of the quenching data with various combinations of Catalán’s polarizability, dipolarity, acidity and basicity parameters [Equation (1)]. The regression results are shown in Table 2. Each entry is the P-value for the coefficient indicated by the column header. The F-value for the overall fit is reported in the right column. P-values greater than 0.05 and F-values less than 3.7 indicate that the correlation with the given parameter is not valid. Of the top eight correlations ranked by the F-values, only the SA term has a valid P-value, with one exception. Adding parameters other than the SA parameter to the regression only makes the correlation worse. There are a few entries with valid P and F-values for the SB term. However, the F-values are significantly smaller, and the P-values significantly greater than the best SA correlation. This analysis indicates that the quenching order of magnitude is best correlated only with the solvent acidity:

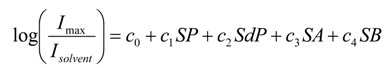
(1)


### 2.2. Preferential Solvation Studies

Several mechanisms could lead to the strong deactivation of the excited states of **1** and **2**. With PRODAN, for example, protonation of the excited state was suggested by calculations as the route for radiationless decay [15]. Another route would be H-bonding-induced deactivation brought on by increasing the coupling with the ground state. Recent preferential solvation studies with the carbony-twisted PRODAN derivatives **3** and **4** have suggested that quenching results from a doubly H-bonding excited state, but not the singly H-bonded excited state. Preferential solvation studies were conducted with **1** and **2** to offer insight into the quenching mechanism.

**Table 2:** Multiple linear regression results for quenching order of magnitude*vs.* various solvent parameter combinations with **1** and **2**. *^a^*

1				2					
SP	SdP	SA	SB	*F*-value	SP	SdP	SA	SB	*F*-value
		2.4 × 10^−6^		193			2.0 × 10^−6^		204
		1.1 × 10^−4^	0.05	170			9.2 × 10^−5^	0.05	175
0.13		3.9 × 10^−6^		127	0.25		1.9 × 10^−4^	0.10	132
	0.13	4.5 × 10^−6^		126	0.15		3.9 × 10^−6^		128
0.14	0.15	0.21	0.07	105		0.15	4.2 × 10^−6^		128
0.78		8.8 × 10^−3^	0.24	96		0.84	3.0 × 10^−3^	0.23	98
	0.97	2.0 × 10^−3^	0.25	94	0.24	0.37	0.02	0.12	87
0.84	0.93	1.2 × 10^−4^		71	0.74	0.47	4.7 × 10^−5^		73
0.01			1.4 × 10^−4^	37	4.7 × 10^−3^			7.2 × 10^−5^	46
			2.6 × 10^−3^	21		0.03		5.2 × 10^−4^	24
	0.07		1.0 × 10^−3^	19				2.7 × 10^−3^	21
0.08	0.07			2	0.29	0.25			1
	0.57			0.4		0.64			0.2
0.90				0.02	0.94				0.006

*^a^* Entries are the *P*-values for the variable of the column heading.

The Rosés and Bosch model for preferential solvation is cast in terms of solvent exchange equilibria as in Equations (2) and (3) [16,17,18,19,20,21,22,23]. However, the model is typically applied to ground state species. In the present case excitation of the fluorophore (*F*) leads to extensive solvent reorganization around the newly created charge-transfer excited state (F*). Because excited states are short-lived, an equilibrium is unlikely to be achieved before deactivation. The equilibrium constants *f*_12/1_ and *f*_2/1_ are actually relative rate constants in this case. These investigators have found that most systems are adequately treated by the simplest model involving just two solvent molecules, either two of the same kind or one of each kind. In Equations (2) and (3), S1 is the aprotic solvent and S2 is ethanol. The rates *f*_12/1_ and *f*_2/1_ are relative to rate of formation of F*(S1)_2_:

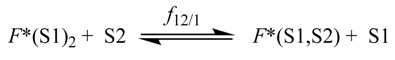
(2)

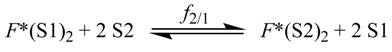
(3)


Preferential solvation is indicated by changes in spectral features that are not linear with changes in the solvent composition. Two spectral values (*Y*) are extracted from each fluorescence spectrum; namely, the relative quantum yield (*Q*_rel_) and the product of the emission center-of-mass and relative quantum yield (*ṽ_CM _* •*Q*_rel_) [24]. The fractional changes (*Γ*) in these values are related to *f*_12/1_ and *f*_2/1_ through Equation (4). The subscripts 1 and 2 refer to the values in the aprotic solvents and in ethanol, respectively, and the x-variable is the ethanol mole fraction. The parameter *r* is the ratio (*Y*_1_ − *Y*_12_)/(*Y*_1_ − *Y*_2_) where *Y*_12_ is the spectral value for the mixed solvent species. The *r*-values, especially *r*_Q_, provide insight into the quenching mechanism:

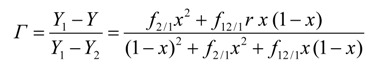
(4)


Two binary mixtures were chosen for preferential solvation titrations: acetonitrile/ethanol (mixture A) and toluene/ethanol (mixture B). The latter mixture is composed of solvents with greatly different polarity values. The SdP value for toluene is 0.284, while that for ethanol is 0.783 [5]. In addition, toluene is not an H-bond donor. In spite of these differences, the absorption spectra are hardly affected by solvent composition. Table 3 shows that the wavelength of maximum absorption shifts 3.7 nm in the most extreme case, **2** in toluene/ethanol. This weak effect suggests that preferential solvation is slight in the ground state.

**Table 3:** Absorption parameters of **1** and **2** in acetonitrile/ethanol and toluene/ethanol mixtures.

	CH_3_CN/EtOH				PhCH_3_/EtOH			
mol%	1		2		1		2	
EtOH	rel ε	λ_max_	rel ε	λ_max_	rel ε	λ_max_	rel ε	λ_max_
0	1.00	374.8	1.00	398.1	1.00	378.1	1.00	398.6
10	0.97	374.8	0.97	398.1	0.97	378.1	0.97	400.0
20	0.97	374.8	0.96	398.1	0.96	378.1	0.94	400.5
40	0.96	374.8	0.95	399.1	0.93	378.1	0.90	401.9
60	0.95	375.3	0.95	399.5	0.91	378.1	0.88	402.3
80	0.95	375.3	0.93	400.0	0.89	377.6	0.86	401.9
90	0.94	375.3	0.94	400.0	0.88	376.7	0.84	401.4
100	0.94	375.3	0.94	400.5	0.88	375.8	0.84	400.5

**Figure 4:**
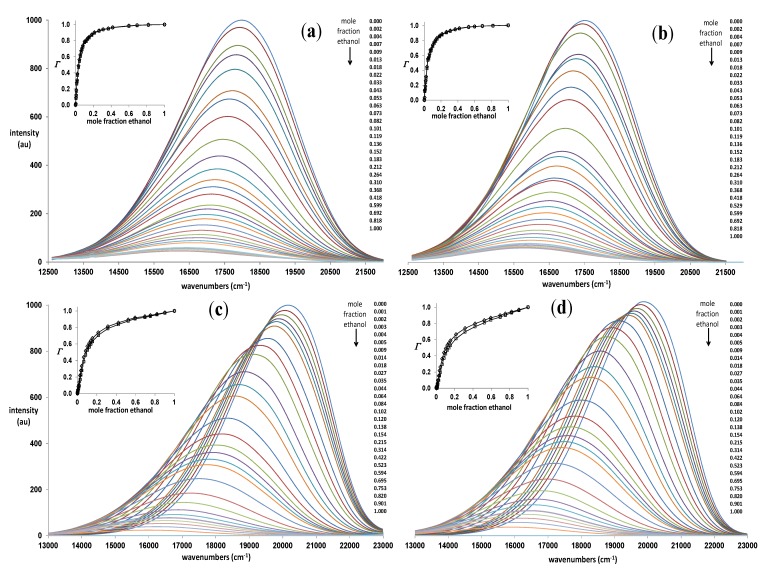
(a) Fluorescence spectra of 1 × 10^−5^ **M** solutions of **1** in acetonitrile/ethanol; (**b**) Fluorescence spectra of 1 × 10^−5^ **M** solutions of **2** in acetonitrile/ethanol; (**c**) Fluorescence spectra of 3 × 10^−5^ **M** solutions of **1** in toluene/ethanol; (**d**) Fluorescence spectra of 2 × 10^−5^ **M** solutions of **2** in toluene/ethanol. Insets: plots of *Γ*_CM∙Q_ (◊) and *Γ*_Q_ (□) *vs.* ethanol mole fraction.

Figure 4 shows that the fluorescence is greatly affected by solvent composition. The plots for mixture A do not show isoemissive points and the inset plots are nearly coincident. Both of these phenomena result from the much smaller shift in the emission center-of-mass (CM) coupled with the more effective quenching by ethanol in acetonitrile. Because there is a large difference in the polarity between toluene and ethanol, the emission CM shifts significantly to lower energy as the mole fraction of ethanol increases. The plots also show that the protic solvent gives rise to extreme quenching. As in the previous study with **3** and **4**, the disappearance of an initial isoemissive point at very low ethanol concentrations points to at least two H-bonded species [25]. The inset plots show clear deviation between *Γ*_CM∙Q_ and *Γ*_Q_. These plots can deviate from each other only when there are more than two emitting species. The plot of the deviation *vs.* mole fraction allows for the determination of the doubly H-bonded solvation factor (*f*_2/1_). With this value, the two plots of *Γ vs.* mole fraction can be fit to provide the singly H-bonded solvation factor (*f*_12/1_) and the *r*-value. The solvation factors are the same for both *Γ* plots, but the *r*-values can differ. The *r*-value is the position of the spectral parameter of the singly H-bonded species relative to the non- and doubly-H-bonded species. That is, an *r*-value that is close to 1.0 indicates that the spectral parameter of the singly-H-bonded species is similar to the doubly H-bonded species, while an *r*-value that is close to 0 indicates similarity to the non-H-bonded species.

The spectral parameters extracted from these titration plots are collected in Table 4. The preferential solvation factors, *f*_12/1_ and *f*_2/1_, are all quite large. The large *f*_12/1_ values manifest themselves in the steep initial slopes of the inset plots (limiting slope = *r* • *f*_12/1_), whereas large *f*_2/1_ values are indicated by the very low ethanol mole fractions where the isoemissive points are lost in the toluene binaries. Both sets of factors are on average 3–5 times greater than those for **3** and **4** in acetonitrile/methanol. These results indicate that the excited states of **1** and **2** show a greater tendency to form H-bonds than **3** and **4**. Quenching in the doubly H-bonded excited states is more efficient than in the singly H-bonded excited states as shown by the *r*_Q_ values all being less than one. All of the singly H-bonded excited states show some quenching, and the extent of quenching is greater with acetonitrile as co-solvent than with toluene. That there is some quenching of the singly H-bonded excited state is in stark contrast to the behavior of excited **3** and **4**. With the latter there is no quenching of the singly H-bonded excited states [25]. Differental quenching of the singly and doubly H-bonded excited states has been postulated with aminofluorenones and aminoanthraquinones [26,27,28]. The difference in the quenching between the singly and doubly H-bonded species can be understood in light of the known quenching behavior of **1** and **2** in aprotic solvents. The excited states of **1** and **2** show energy-gap dependent quenching typical of charge-transfer states [29,30]. That is, as the energy gap between the solvent-stabilized excited state and the ground state decreases, radiationless deactivation increases and the fluorescence intensity decreases [13]. Since H-bonding also shifts the emission to lower energy, the quenching induced by H-bonding can result from the smaller energy gap or from the increased coupling with the ground state or more likely a combination of both. With the toluene/ethanol system, the shifts in the emission CM (*r*_CM_ = 0.21 and 0.26 for **1** and **2**, resp.) are greater than the decrease in the quantum yields (*r*_Q_ = 0.11 and 0.17 for **1** and **2**, resp.) for the singly H-bonded excited states. The decrease in the energy gap with the ground state due to H-bonding may be sufficient to explain the quenching in this case. On the other hand with the acetonitrile/ethanol system, the change in the emission CM is no more than double on an absolute scale that of the toluene/ethanol system, while the quenching is 4–5 times greater. Quenching here must be due to enhanced coupling, but the enhanced coupling is made possible by the already reduced energy gap with this highly polar binary system. Energy-gap dependent fluorescence quenching induced by intermolecular H-bonds has been proposed by Han and co-workers for fluorenone derivatives [31,32].

For derivatives **3** and **4** the H-bond quenching mechanism is turned on by the twisting of the carbonyl groups out of the plane of the naphthalene rings. The deviation from planarity carries over to the excited states, but to a lesser extent. Planar derivatives and PRODAN itself do not show significant quenching in protic solvents. Here the excited-states show steeper barriers to rotation out of the naphthalene plane [33]. Previous calculations show that both **1** and **2** show similar out-of-plane twisting, which is due to steric interactions with the peri-H in these molecules. The excited state of **1** is still calculated to be twisted out-of-plane, whereas the excited state of **2** is predicted to be planar, but to have essentially no barrier (<1 kcal/mol) for out-of-plane deviations through 30°. Thus, H-bonding with twisted or easily twisted carbonyl groups appears to offer a deactivation pathway for **1** and **2**.

**Table 4:** Calculated preferential solvation parameters for **1** and **2** in acetonitrile/ethanol (A) and toluene/ethanol (B) mixtures. *^a^*

	mixture	*f*_12/1_	*f*_2/1_	*Q*_12_ *^b^*	*Q*_2_	*r*_Q_ *^c^*	*r*_CM_ *^d^*	Δ*ṽ ^e^*
**1**	A	41	550	0.44	0.07	0.59	0.64	2400
		(4)	(20)	(0.03)	(0.03)	(0.02)	(0.02)	(800)
**2**	A	32	270	0.36	0.08	0.69	0.74	1900
		(4)	(110)	(0.12)	(0.01)	(0.12)	(0.12)	(100)
**1**	B	44	460	0.90	0.05	0.11	0.21	4400
		(1)	(100)	(0.02)	(0.01)	(0.02)	(0.02)	(100)
**2**	B	50	250	0.84	0.08	0.17	0.26	4500
		(9)	(50)	(0.07)	(0.01)	(0.07)	(0.06)	(200)

*^a^* Parenthetical values are standard deviations of multiple experiments; *^b^ Q*_1 _~ 1.0; *^c^* *r*_Q_ = (*Q*_1_ − *Q*_12_)/(*Q*_1_ − *Q*_2_); *^d^* *r*_CM_ = (*ṽ_CM_*_(1)_ − *ṽ_CM_*_(12)_)/(*ṽ_CM_*_(1)_ − *ṽ_CM_*_(2)_); *^e^* Δ*ṽ* = *ṽ_CM_*_(1)_ − *ṽ_CM_*_(2)_.

## 3. Experimental

### 3.1. General Methods

Fluorescence emission data were collected using a fiber optic system with 405 nm LED light source and a high sensitivity Ocean Optics Maya CCD detector (Ocean Optics, Dunedin, FL, USA) in a chamber thermostated at 23 °C. Absorption spectra were obtained from the same fiber optic system with a miniature deuterium/tungsten light source. Solvents used for photophysical characterization are 99% (2-butanol, 1-decanol, 1-pentanol), HPLC grade (*t*-butanol, 1-octanol, 1-propanol) or spectrophotometric grade (isopropanol, 1-butanol, ethanol). Compounds **1** and **2** were available from a previous study [13].

### 3.2. Solvent Acidity Studies

#### 3.2.1. Absorption Data

Relative molar absorptivities were determined by the method of standard additions. To each cell containing 2.0 mL of an alcohol was added four equal aliquots (5–10 μL) of a stock solutions of **1** and **2** in toluene. The lamp transmission was recorded for the blank and after addition of each aliquot. After subtracting the background (dark) from each transmission value, the value of log(I_o_/I) at 405 nm was determined after each aliquot addition. The absorbance of the first aliquot solution was determined from the slope of the line of the plot of log(I_o_/I_x_) *vs.* x = 1,2,3,4 forcing a 0-intercept.

#### 3.2.2. Fluorescence Data

Solutions were prepared by diluting 20 μL of a stock solution of the 2,5-PRODAN derivative (~10 mg/10 mL isopropanol) to 2.00 mL with the following series of nine alcohols: *t*-butanol, 2-butanol, 1-decanol, isopropanol, 1-octanol, 1-pentanol, 1-butanol, 1-propanol and ethanol. The instrument data was manipulated as follows. The abscissa scale was transformed to wavenumbers, and the intensity at each point was multiplied by λ^2^ to account for the effect of the diffraction grating [14]. These values were divided by the spectral response of the Hamamatsu S10420 CCD at each point. The electronic noise, determined from the dark spectra, was subtracted from the adjusted emission intensities before numerical integration. Integrated emission intensities (*I* *=* ∫*I(ṽ) dṽ*) were adjusted by the relative molar absorptivities (*ε*) and refractive indices (*η*) in each solvent: *I*_solvent_ = *I* *•* (*ε*_max_/*ε*)* •* (*η*^2^/*η*^2^_min_).

### 3.3. Preferential Solvation Studies

#### 3.3.1. Determination of Spectral Values (*Y*) and Fractional Changes (*Γ*)

Solutions of identical concentrations of the fluorophore were made by diluting 25 μL of the stock solution to 5 mL with the two solvents of interest. Two sets of emission data were acquired for each binary mixture study. The first set begins with a 2.0 mL sample of the solution in the aprotic solvent (toluene or acetonitrile). To this sample up to 28 aliquots of the ethanol solution were sequentially added, and the emission spectrum was recorded after mixing for one minute. In the second set, the initial sample was 2.0 mL of the solution in ethanol, and four aliquots of the solution in the aprotic solvent were added. Absorption spectra were determined in binary mixtures ranging from 0% to 100% using the method of standard additions above. The relative molar absorptivities at 405 nm of the binary mixtures were estimated from the best third-order polynomial-fit to the plot of normalized absorbance *vs.* mole fraction ethanol. The refractive indices of the toluene-ethanol mixtures were interpolated from experimental data [34]. Refractive indices for the acetonitrile-ethanol mixtures were calculated using the Gladestone-Dale equation [35] using density data [36] for known solvent compositions. Integrated emission intensities were adjusted by *ε* and *η* using the method above. The emission center-of-mass (*ṽ_CM_*) was determined from the following quotient: ∫*I(ṽ)•ṽ dṽ* ∕ ∫*I(ṽ) dṽ.* Both the relative quantum yield, *Q*_rel_ = *I/I*_max_, and the product of the center-of-mass and the relative quantum yield, *ṽ_CM _*•*Q*_rel_ [24] were determined for each fluorescence spectrum. The fractional change *Γ* in the spectral values (*Y,* either *Q*_rel_ or *ṽ_CM _*•*Q*_rel_) from those in the aprotic solvent are calculated from the expression *Γ* = (*Y*_1_ − *Y*)/(*Y*_1_ − *Y*_2_) where *Y*_1_ and *Y*_2_ are the spectral values in the pure aprotic and protic solvent, respectively, and *Y* is the spectral value for a given binary solvent mixture.

#### 3.3.2. Determination of preferential solvation parameters

Both sets of fractional changes, *Γ_Q_* and *Γ_CM•Q_*, are governed by the same relative rates *f*_2/1_ and *f*_12/1_. The *f*_2/1 _values are determined by finding the mole fraction where the plot of *Γ_CM•Q_* - *Γ_Q_ vs.* mole fraction achieves a maximum through this equation: *f*_2/1_ = *x*^−2^ − 2*x*^−1^ + 1 [25]. This maximum is determined by fitting six or more points around the maximum to a third-order polynomial function, taking its derivative, setting it to zero and solving for the mole fraction. The values for *f*_12/1_ and *r* are determined through non-linear least squares fitting of the plots of *Γ_CM•Q_* and *Γ_Q_ vs.* mole fraction using the value for *f*_2/1_ obtained above.

## 4. Conclusions

Compounds **1** and **2**, both 2,5-regioisomers of PRODAN, show strong quenching by alcohols. The magnitude of the quenching is more than double that of PRODAN derivatives **3** and **4**. All four compounds share the commonality of a structure where the carbonyl group is twisted or easily twisted out of the molecular plane of the naphthalene ring. It is thought that this twisting turns on coupling between the H-bonded excited state with the ground state leading to efficient deactivation. This coupling is very efficient with two H-bonds, and partially efficient with one H-bond when the other co-solvent is polar. The strong quenching makes **1** and **2** sensitive sensors of solvent acidity for weakly acidic solvents.
